# "Research protocol: a synthesis of qualitative studies on the process of adaptation to dependency in elderly persons and their families"

**DOI:** 10.1186/1471-2318-10-58

**Published:** 2010-08-25

**Authors:** Eva Abad-Corpa, Teresa González-Gil, Ana M Barderas-Manchado, Carmen de la Cuesta-Benjumea, Olga Monistrol-Ruano, Vinita Mahtani-Chugani, Antonio Martínez-Hernández

**Affiliations:** 1 Research Department, Zone VI Management Vega Media del Segura, C/Marqués de los Vélez s/n, 30008 Murcia, Spain; 2 Nursing Department, Health Science Faculty, Rey Juan Carlos University, Avda de Atenas s/n, 28922, Alcorcón, Madrid, Spain; 3 Health Research Foundation, Carlos III Health Institute, Spanish Ministry of Science and Innovation, C/Monforte de Lemos 5, 28029, Madrid, Spain; 4 Department of Nursing, University of Alicante, Carretera San Vicente del Raspeig s/n, 03690 San Vicente del Raspeig, Alicante, Spain; 5 Nursing Research Area, Mútua de Terrassa University Hospital, Plaça Dr. Robert 5, 08221 Terrassa, Spain; 6 Canary Islands Health Care Services, Primary Care Management Tenerife, C/Carmen Monteverde nº 45, 2ª planta, 38003 Santa Cruz de Tenerife, Spain; 7 Spanish Network for Research in Epidemiology and Public Health (CIBERESP), Spain; 8 Murcia Health Service. Dirección General de Planificación, Ordenación Sanitaria y Farmacéutica e Investigación. C/Pinares 6, 4ª planta, 30001 Murcia. Spain; 9 Carlos III Health Institute. Nursing Research Coordination and Development Unit (Investen-isciii). C/Monforte de Lemos, 5. 28029 Madrid, Spain

## Abstract

**Background:**

Dealing with dependency in the elderly and their families leads us to explore the life experience of those involved together with the processes of adaptation to this condition. A number of original studies have been published which, following a qualitative methodology, have dealt with both dimensions.

**Methods/Design:**

**Objectives**: 1) To present a synthesis of the qualitative evidence available on the process of adaptation to dependency in elderly persons and their families; 2) to conduct an in-depth study into the experiences and strategies developed by both to optimise their living conditions; 3) to enable standards of action/intervention to be developed in the caregiving environment.

A synthesis of qualitative studies is projected with an extensive and inclusive bibliography search strategy. The primary search will focus on the major databases (CINAHL, MEDLINE, EMBASE, PsycInfo, PSICODOC, Cochrane Library, JBI, EMBASE, LILACS, CUIDEN, CUIDEN qualitative, CUIDATGE, British Nursing Index, SSCI). The secondary search will be conducted in articles taken from the references to studies identified in the articles and reports and the manual search in congresses and foundation papers. Article quality will be assessed by the guide proposed by Sandelowski & Barroso and data extraction done using the QARI data extraction form proposed by the Joanna Briggs Institute for Evidence-Based Practice.

The synthesis of the findings will be based on the principles and procedures of grounded theory: coding, identification and relationship between categories, and synthesis using constant comparison as a strategy.

**Discussion:**

This synthesis of qualitative evidence will enable us to detect health needs as perceived by the receivers in their own interaction contexts.

## Background

Europe is currently, after Japan, the most aged region worldwide, with 16% of the population aged over 65 years [1]. International predictions also conclude that this ageing will increase more rapidly than in any other region in the world [2].

According to these international predictions, Spain (where the present study is projected) will have the oldest population in the world by 2050 [1]. This ageing process is steadily increasing the number of persons in situations of dependency, with the over 65s comprising three quarters of the total number of dependent persons (despite studies claiming that most basic activities of daily living are maintained beyond the age of 73 [3]). It could be estimated that almost a million and a half elderly persons are in a situation of dependency in Spain, of whom more than 200,000 have absolute dependency and more than 400,000 serious dependency [4]. Taking current reality and trends into account we can expect for a predictable future an increase in the number of elderly persons who are older and frailer than in previous decades [5]. The above situation will obviously have a serious effect on the socioeconomic and health aspect of the community (with the elderly person and family taking on special significance). As far as the family is concerned, it should be noted that population ageing together with advances in medicine have made chronicity a normal issue and home care something *natural *in family life. Moreover, the family is nowadays considered the major healthcare resource; in fact community care policies for the elderly have increased dependency on the family for caregiving [6]. Family caregiving has thus become an expanding phenomenon, attracting the attention of policy-makers and providers of formal support services [7].

This demographic tendency towards a greater longevity means that the quality of life reclaimed from death becomes the focal point in various contexts, particularly the socio-sanitary context. The evolution of disability and dependency, and how to deal with them, is thus a priority issue in the different areas of caregiving.

Disability is understood as the inability to carry out activities of daily living and the need for partial or total assistance to perform them. Disability in the elderly (those aged over 65) is the result of physiological changes related to age, chronic diseases and acute or intercurrent processes [4,8]. And all this influenced by setting.

Closely related to the concept of disability, and in keeping with the major interest it generates within the scientific community, is the concept of frailty, understood as a situation of reduced capacity for response to possible stress-generating agents [9] (including physiological, clinical, functional, psychological and social factors).

Dependency, on the other hand, is the social consequence of disability and frailty. The "dependent elderly person" might therefore be defined as someone who, for reasons of acquired disability and due to his or her inability to cope with this disability, has a permanent loss of physical, mental or intellectual autonomy for carrying out activities of daily living and requires the attention of another person or persons or significant assistance.

The issue of how to deal with dependency leads us to explore people's experience with dependency and related practices of traditional caregiving. A number of original articles have been published, which, following a qualitative methodology, have dealt with both dimensions: experiences and processes of adaptation.

As for people's experience regarding dependency, various investigations have attempted to identify the characteristics and attributes of the concept of disability, frailty and dependency as construed by the elderly themselves and their carers [10-12]. Other studies focus on the process of coping with dependency and on the identification of strategies developed by the elderly to adapt to their situation [13] by redefining the concepts of autonomy and control [14].

Taking into account the different causes of disability in elderly persons, there are studies that deal with the experience of the elderly and their carers in a variety of contexts. These contexts would be related to: chronic diseases [15], with special emphasis on pain [16-18]; acute problems (transitory disability) [19,20]; problems of mental health (especially dementia) [21-23]; and the limitations typical of ageing [24,25].

Accordingly, and in keeping with this important research output, the present research proposal advocates a synthesis of existing qualitative studies to gather available qualitative evidence and enable standards of action/intervention to be developed in the caregiving environment.

This synthesis of qualitative evidence will enable us to detect health needs as perceived by the receivers in their own interaction contexts. This will give us access to a much deeper, more real, more integral and more holistic knowledge of our field of action (as carers), in order to guide us towards a better-quality practice. Furthermore, considering the voice of the community (in this case the elderly and their families) enables their active role within the caregiving system to be recognised, i.e. their power of decision-making.

Accordingly, the following research objectives are proposed:

(a) General:

To present a synthesis of qualitative evidence on the process of adaptation to dependency in elderly persons and their families.

b) Specific:

- To identify the components that give meaning to the concept of dependency in elderly persons and their families.

- To understand the experience and process of change/adaptation to dependency in elderly persons and their families.

- To identify strategies developed by elderly persons and their families to optimise their living conditions.

- To assess the overall scientific quality of the qualitative studies encountered on the subject.

## Methods/Design

### Design

A synthesis of qualitative studies is proposed.

### Type of studies

Studies will be included that specify any qualitative method including different types concerning the object of study such as life histories, phenomenological studies, grounded theory and/or ethnographic studies.

### Type of participants

The synthesis will include studies on participants aged over 65 years with any type of physical or mental dependency and at any time of the vital process (acute, chronic, recent, years of evolution, etc.).

Likewise it will include studies in which participants are relatives of elderly persons (in the above situations) and act as carers.

It will exclude studies whose participants are in the sphere of patients in a terminal situation due to the singular characteristics of their life situation.

### Type of interventions/phenomena of interest

The synthesis will include studies where the phenomenon of interest is a description and interpretation of the experience of elderly persons and their families in the process of adaptation to dependency and the strategies developed to optimise their living conditions.

### Type of outcomes studied

The outcomes of interest are the own experiences of the elderly and their families, including feelings such as loss, blame, hope, disconnection, unreality, control, etc.

### Search strategies for studies

The bibliography search will be extensive and inclusive and will include both a search of published studies indexed in international bibliography databases and a search of unpublished studies and interviews with key persons (contributing their in-depth knowledge on the location of qualitative studies).

A strategy will thus be carried out in three stages.

- The first stage will conduct an automated search in the CINAHL, MEDLINE and EMBASE databases of publications over the last two years to analyse the words contained in the title, abstract and MeSh terms of the most relevant articles on the subject to allow identification of additional search terms. This initial search will use the filters established for seeking qualitative studies in CINAHL, EMBASE and adaptations to MEDLINE.

- The second stage will again conduct an electronic search with the incorporation of new terms (detected in the first stage) and specific key words for each of the databases.

The search will include studies published in English, French, Spanish and Portuguese, with no limit in time, in the following databases: CINAHL, Medline, PsycInfo, PSICODOC, Cochrane Library, JBI, EMBASE, LILACS, CUIDEN, CUIDEN qualitative, CUIDATGE, British Nursing Index, Social Science Citation Index (SSCI).

- The third and final stage will consist of a secondary search of articles taken from the references of studies identified in the articles and reports and a manual search in congresses, foundation papers, etc.

### Selection of studies

Two observers will independently and blindly assess the titles and abstracts of the identified references to evaluate their potential eligibility. The studies will include whether they fulfil all the abovementioned criteria with regard to: 1) Type of studies; 2) Type of participants; 3) Interventions/phenomena studied; 4) Outcomes studied.

### Assessment of the methodological quality of studies

Critical reading and assessment of the quality of the studies will follow the guide proposed by Sandelowski & Barroso [26].

A pilot trial will be conducted to assess the feasibility of the use of this guide and evaluate the homogeneity of data collection.

The methodological quality of the articles will be assessed independently and blindly by two reviewers. In the event of discrepancies an attempt will be made to reach a consensus and where not possible the decision will be considered by a third reviewer.

### Data extraction

Data extraction will be done using the template contemplated by the QARI data extraction tool proposed by the Joanna Briggs Institute for Evidence-Based Practice. A pilot trial of data extraction will also be conducted to check the adequacy of the form and optimise it if necessary. Two reviewers will independently obtain and record the data and any discrepancy between them will be resolved by discussion with or the intervention of a third reviewer if necessary.

Should it be necessary the principal author of the study will be contacted to obtain information not available in the publication.

### Data synthesis

Analysis of the evidence will be based on the principles and procedures of grounded theory [27]: coding, identification and relationship between categories, and synthesis using constant comparison as a strategy.

The software package used for data organisation will be the Qualitative Assessment Review Instrument" (QARI) proposed by the Joanna Briggs Institute for Evidence-Based Practice [28].

### Limitations

- It would be interesting to be able to include studies conducted in non-English-, -French-, -Spanish- or -Portuguese-speaking countries; however, studies published in other languages will probably refer to populations with very particular sociocultural contexts;

- The variable methodological quality of the studies may make synthesis of the results difficult;

- Difficult access to unpublished information in journals with a wide circulation. For this reason the manual search will be articulated.

## Discussion

The process of population ageing is steadily increasing the number of elderly persons in a situation of dependency, with the over 65s representing three quarters of the total number of dependent persons. It is therefore very important for the dependency effect to have minimum repercussions on the person and his or her family, which is why we healthcare professionals undergo a wide spectrum of training, support, etc.

The present qualitative synthesis aims to portray the most accurate interpretation of the process of adaptation to dependency and to compare and contrast the constructs of individual studies and their contexts in an attempt to generate a theory that groups the outcomes of these studies on the construction of the phenomenon studied.

Identifying qualitative evidence, therefore, will be highly useful for reaching a consensus on care policies in keeping with community needs and the tendency towards community empowerment.

## Competing interests

The authors declare that they have no competing interests.

## Authors' contributions

EAC: Study conception and design; drafting of manuscript; read and approved the final manuscript; obtaining of funding. TGG: Study conception and design; drafting of manuscript; read and approved the final manuscript; obtaining of funding. ABM: Study conception and design; bibliography search; read and approved the final manuscript; obtaining of funding. CCB: Study design; read and approved the final manuscript; obtaining of funding. OMR: Study design; read and approved the final manuscript; obtaining of funding. VMC: Study design; read and approved the final manuscript; obtaining of funding. AMH: Drafting of manuscript; read and approved the final manuscript; administrative, technical and material support. Group RETICEF-evidencia: Read and approved the final manuscript; obtaining of funding.

## Pre-publication history

The pre-publication history for this paper can be accessed here:

http://www.biomedcentral.com/1471-2318/10/58/prepub
